# Supplementary material for: Three-dimensional surface motion capture of multiple freely moving pigs using MAMMAL
Source: Nat Commun. 2023 Nov 25;14:7727. doi: 10.1038/s41467-023-43483-w (PMC10673844; doi:10.1038/s41467-023-43483-w)
Supplement: Supplementary file 1 — Supplementary Information [file 41467_2023_43483_MOESM1_ESM.pdf]

Supplementary Information

Three-dimensional surface motion capture of multiple freely moving pigs using MAMMAL

Liang An[1], Jilong Ren[2,3], Tao Yu[1,4], Tang Hai[2,3*], Yichang Jia[5,6,7*], and Yebin Liu[1,8*]

[1]Department of Automation, Tsinghua University, Beijing, China
[2]State Key Laboratory of Stem Cell and Reproductive Biology, Institute of Zoology, Chinese Academy of Sciences, Beijing, China
[3]Beijing Farm Animal Research Center, Institute of Zoology, Chinese Academy of Sciences, Beijing, China
[4]Tsinghua University Beijing National Research Center for Information Science and Technology (BNRist), Beijing, China
[5]School of Medicine, Tsinghua University, Beijing, China
[6]IDG/McGovern Institute for Brain Research at Tsinghua, Beijing, China
[7]Tsinghua Laboratory of Brain and Intelligence, Beijing, China
[8]Institute for Brain and Cognitive Sciences, Tsinghua University, Beijing, China

* Corresponding author
Please address correspondence to:
Tang Hai, Ph.D.
State Key Laboratory of Stem Cell and Reproductive Biology, Institute of Zoology, Chinese Academy of Sciences, Beijing, China
Email: haitang@ioz.ac.cn
Yichang Jia, Ph. D.
School of Medicine, Medical Science Building, Room D204, Tsinghua University, Beijing, 100084, P. R. China
Tel: 86-10-62781045, Email: yichangjia@tsinghua.edu.cn
Yebin Liu, Ph. D.
Central Main Building, Room 825, Tsinghua University, Beijing, 100084, P. R. China
Tel: 86-10-62788613, Email: liuyebin@mail.tsinghua.edu.cn

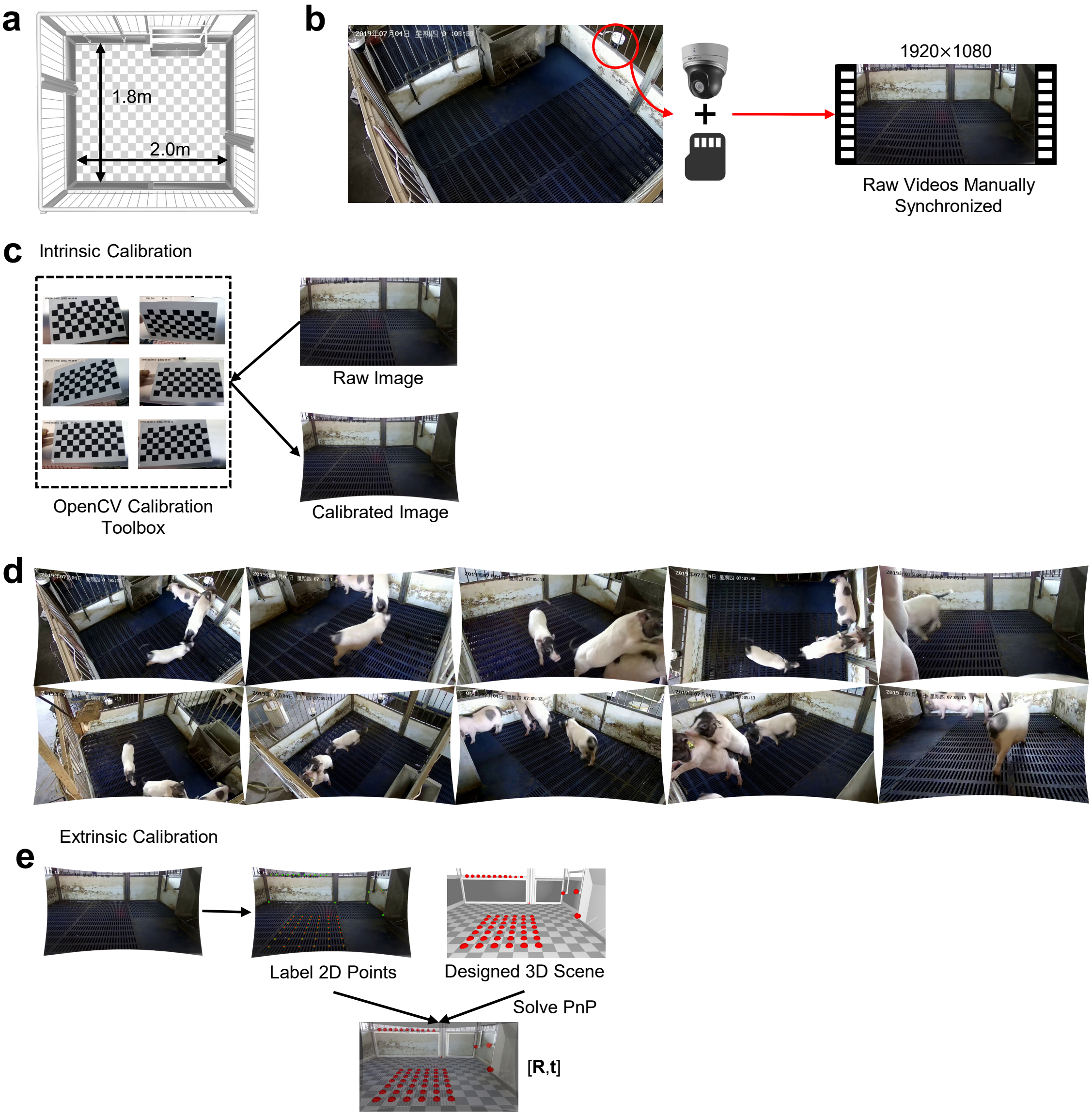

Supplementary Figure 1. 2D video data collection and camera calibration. **a**, Our setting (2.0m × 1.8m) for pig (4 pigs) housing and social interaction. **b**, Ten network cameras with SD cards were installed around the cage. The videos were synchronized manually with 1920 × 1080 pixel resolution. **c**, Intrinsic calibration using chessboard images and OpenCV calibration toolbox transforms raw video images to undistorted ones. Note that all cameras share the same intrinsic parameters; therefore, we employed the same parameters for all the cameras. **d**, Illustration of the 10 input images after intrinsic calibration. **e**, The extrinsic calibration process utilized manually labeled scene points to solve the projective n-points (PnP), yielding the rotation and translation of each camera view.

a

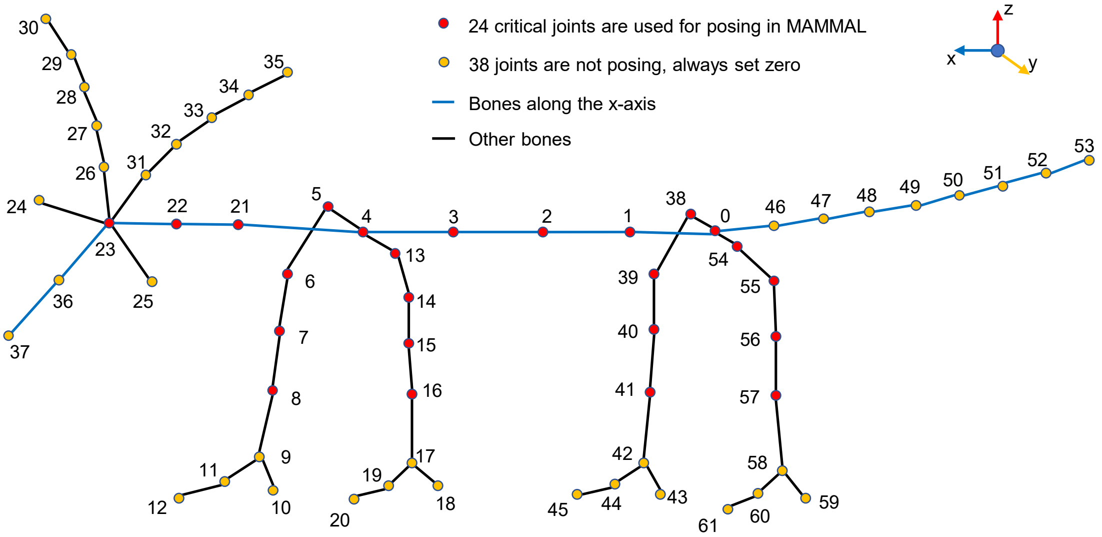

b

Left arm		Right arm		Left leg		Right leg		Tail		Main body	
13	LF hip	5	RF hip	54	L leg	38	R leg	46	Tail1	0	Hip
14	LF clavicle	6	RF clavicle	55	L leg	39	R leg	47	Tail2	1	Spine1
15	LF leg	7	RF leg	56	L knee	40	R knee	48	Tail3	2	Spine2
16	LF knee	8	RF knee	57	L foot	41	R foot	49	Tail4	3	Spine3
17	LF foot	9	RF foot	58	L foot base	42	R foot base	50	Tail5	4	Front hip
18	LF heel	10	RF heel	59	L heel	43	R heel	51	Tail6	21	Neck1
19	LF toe base	11	RF toe base	60	L ball back	44	R ball back	52	Tail7	22	Neck2
20	LF toe end	12	RF toe end	61	L toe	45	R toe	53	Tail8		

Head				Left ear				Right ear			
23	Head	36	Jaw	31	L ear	34	L ear3	26	R ear	29	R ear3
24	R eye	37	Jaw end	32	L ear1	35	L ear4	27	R ear1	30	R ear4
25	L eye			33	L ear2			28	R ear2		
			Note: 'R' means Right, 'L' means Left, 'LF' means Left Front, 'RF' means Right Front								

c

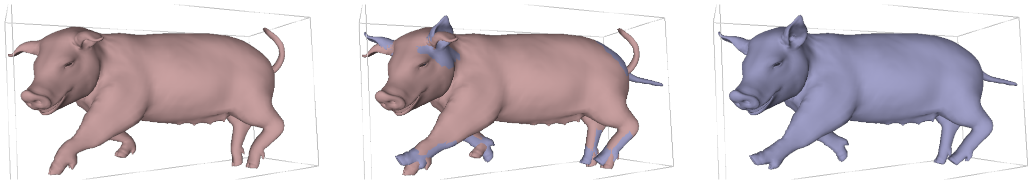

Animation of 62 joints — Comparison — Animation of 24 crucial joints

Supplementary Figure 2. Joints and crucial joints of the PIG model. **a** and **b,** The topological structure (**a**) and names (**b**) of 62 PIG joints. The joints at rest pose are symmetric about y-axis and face along x-axis. **c**, Comparison between the animations driven by 62 joints and 24 crucial joints. Currently, the 24 crucial joints ignore subtle motions on ears, toes, jaw and tail, which have limited influence for locomotion and social behavior analysis.

a **b**

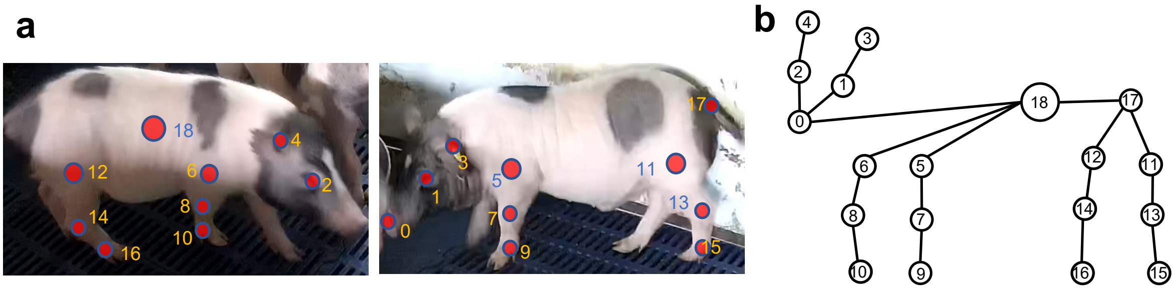

c

Id	Name	Id	Name	Id	Name	Id	Name
0	Nose	5	Left Shoulder	10	Right Paw	15	Left Foot
1	Left Eye	6	Right Shoulder	11	Left Hip	16	Right Foot
2	Right Eye	7	Left Elbow	12	Right Hip	17	Tail
3	Left Ear	8	Right Elbow	13	Left Knee	18	Center
4	Right Ear	9	Left Paw	14	Right Knee		

Supplementary Figure 3. Definition of 2D keypoints. **a**, Illustration of 2D keypoints that were referenced by a previous annotation of human 2D keypoints (PMID: 31331883 [https://doi.org/10.1109/TPAMI.2019.2929257]). The size of circle reflects the extent of labeling ambiguity. **b** and **c**, Topological positions (**b**) and names (**c**) of pig 19 keypoints.

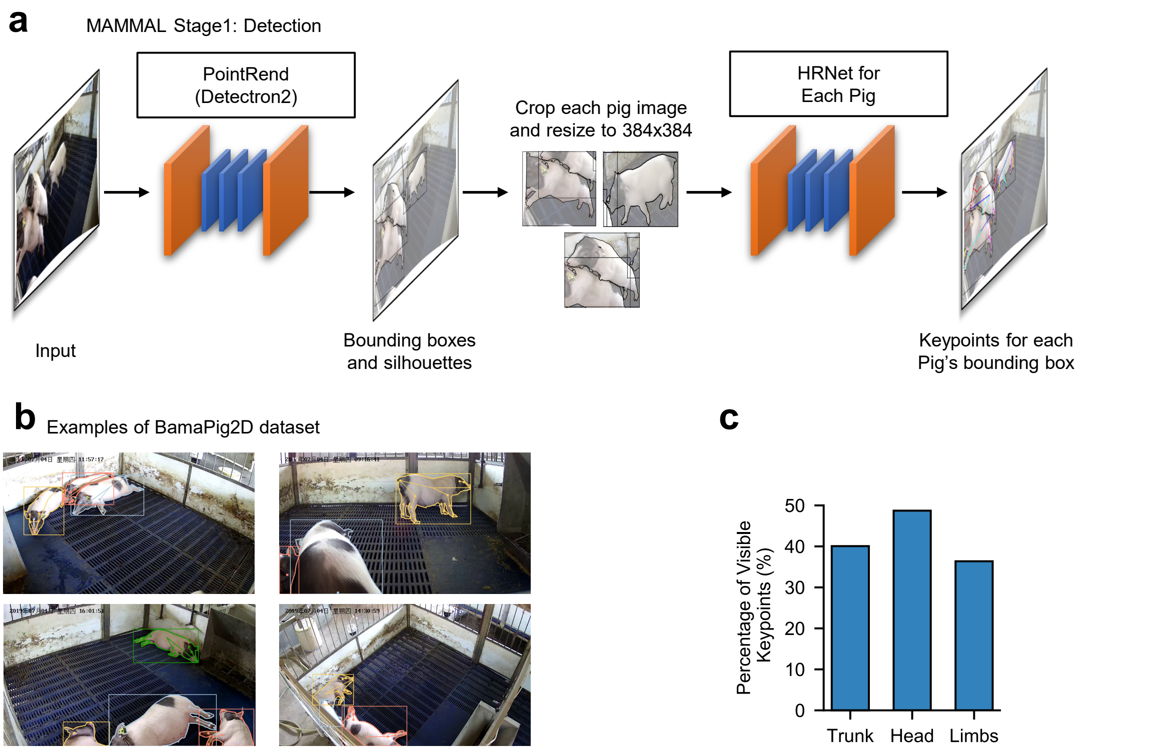

Supplementary Figure 4. 2D pose detection and BamaPig2D dataset. **a**, At MAMMAL Detection stage, the PointRend method was applied to the images to yield bounding boxes and silhouettes. Then, the cropped images of each pig were fed into HRNet to produce keypoints. **b**, Examples of manually-annotated images (3340 in total) of the BamaPig2D dataset. Note that only visible keypoints are labeled. **c**, Percentage of visible keypoints within the BamaPig2D dataset. Keypoints: trunk (0, 17, and 18), head (1, 2, 3, and 4), limbs (5-16).

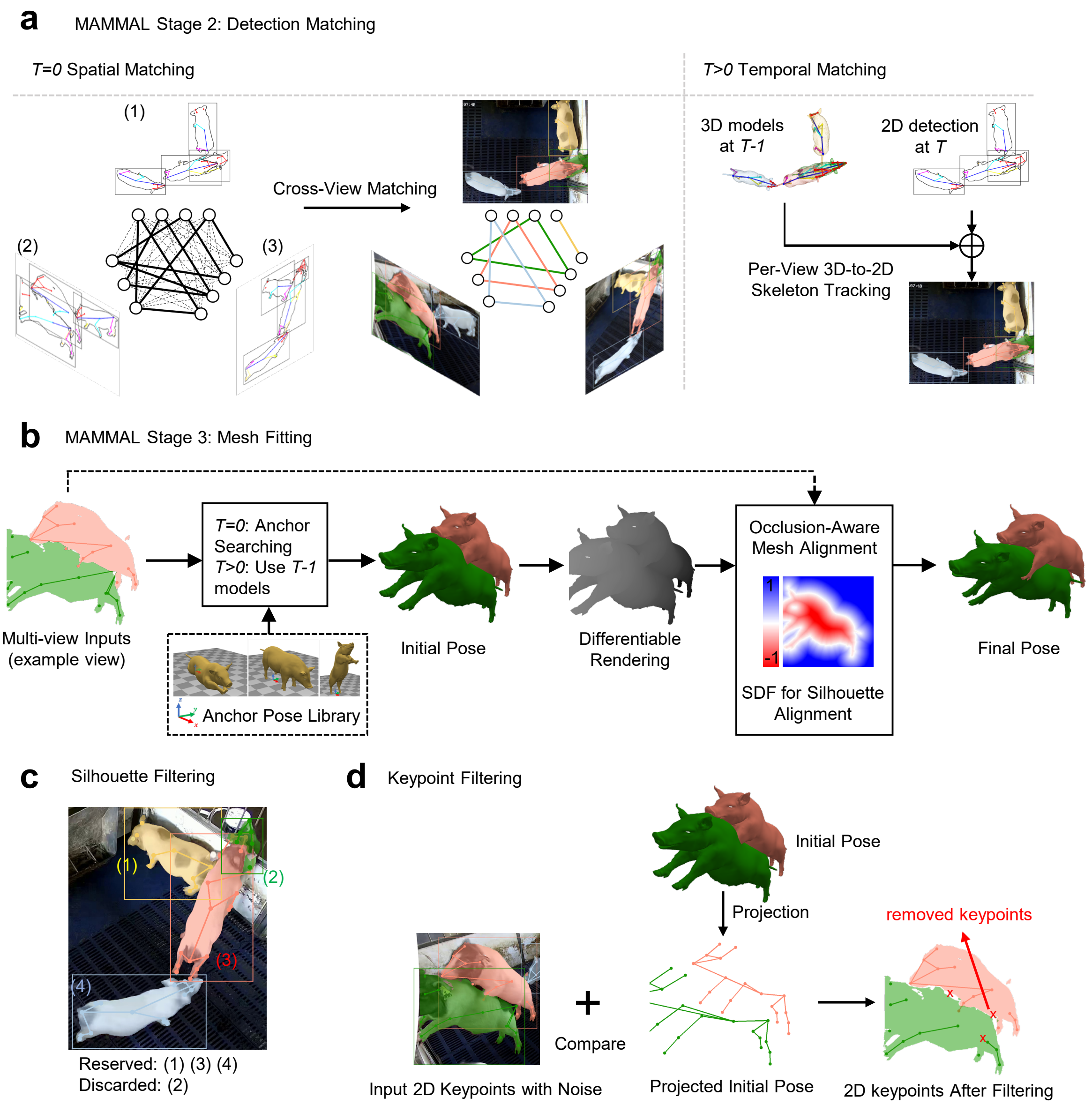

Supplementary Figure 5. The second and third stages of MAMMAL pipeline. **a**, Illustration of the **Detection Matching** of MAMMAL. Left, a 3D association graph was constructed across views to obtain view-consistent 2D detection clusters. Right, temporal tracking is performed on each view to efficiently obtain 2D detection clusters. *T*, current time point of the given image; *T-1*, previous time point 0.04s prior to *T*. **b**, Illustration of the Mesh Fitting of MAMMAL. By taking multi-view detection as inputs, MAMMAL first obtains initial pose estimation by searching the anchor pose library or inherit poses from *T-1* and then performs occlusion-aware mesh alignment using differentiable rendering to yield final poses. **c**, During Mesh Fitting, we filtered out small broken silhouettes severely occluded. **d**, During Mesh Fitting, we filtered out incorrect 2D keypoints which often appeared in occlusion cases by comparing them to the projected initial pose.

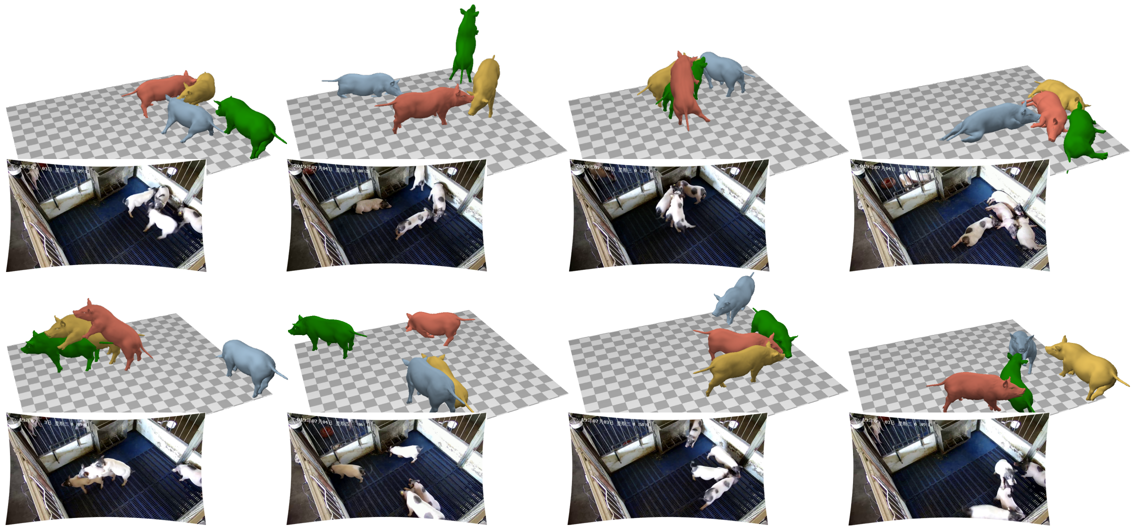

Supplementary Figure 6. More qualitative social pose reconstruction results by MAMMAL. These results are social pose reconstruction at eight time points with no temporal information engaged (set *T*=0 for every time point). Complex behaviors, including three-pig mounting (left bottom) or attack behavior (right bottom), can be reconstructed.

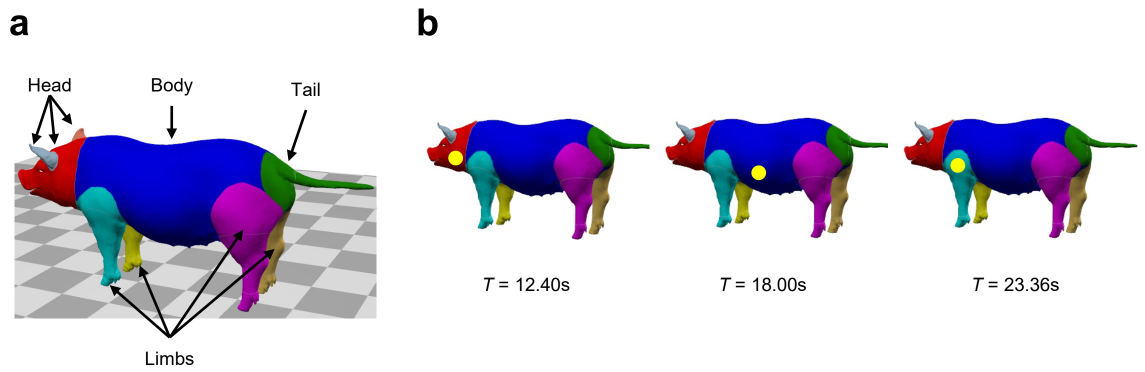

Supplementary Figure 7. Body part definition on pig surface. **a,** The whole body surface is divided into 4 main parts for social behavior analysis. **b,** The yellow circles indicate contact points at each time point of the sequence in Fig. 2g.

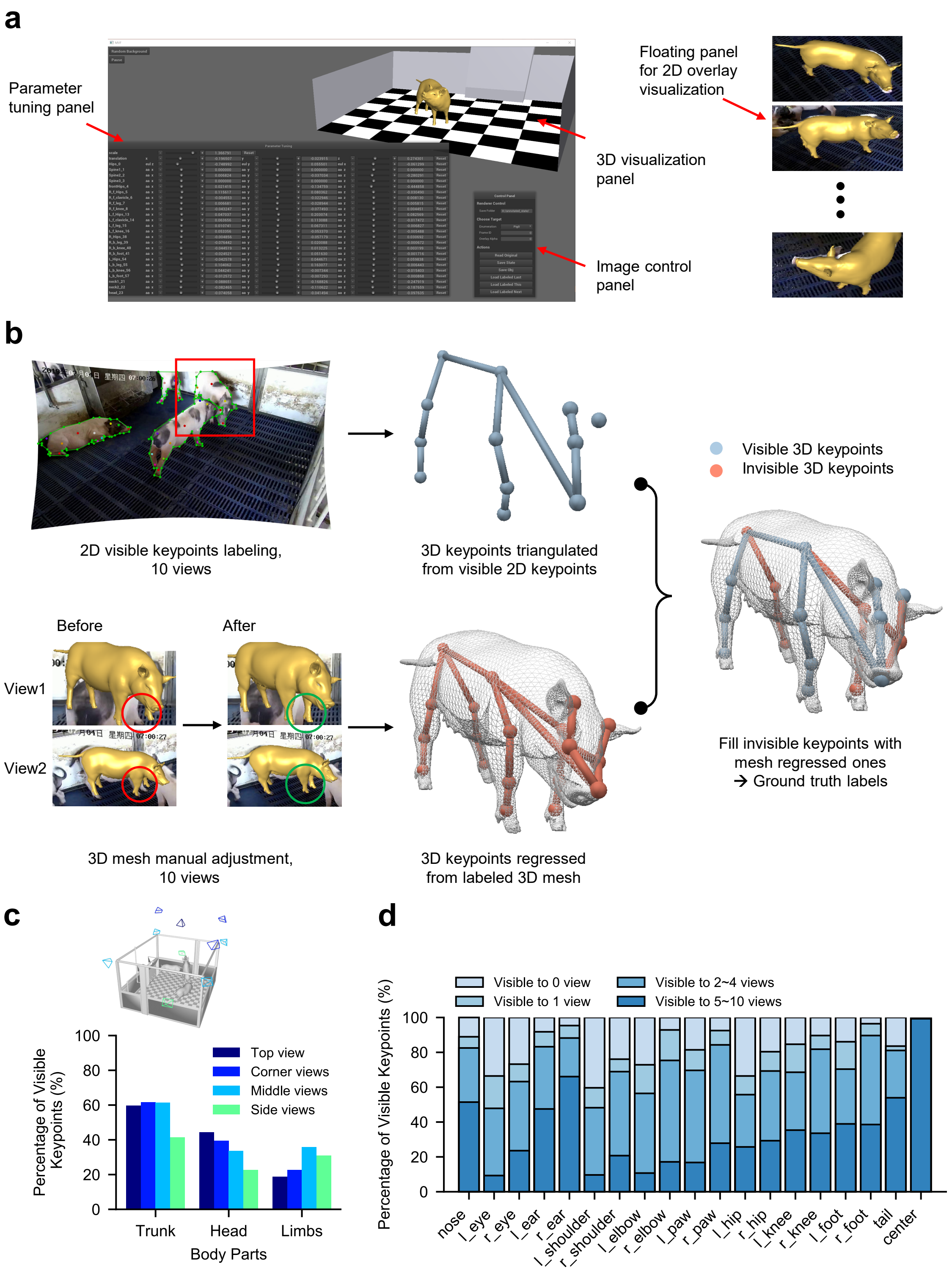

Supplementary Figure 8. Software and pipeline for BamaPig3D dataset annotation. (Figure legend on next page).

Supplementary Figure 8. Software and pipeline for BamaPig3D dataset annotation. a, The graphics user interface (GUI) of our designed software for pig pose adjustment containing four panels: 1) A parameter tuning panel for pose of a given pig; 2) A 3D visualization panel for visualizing the tuned pose in real time, and the annotator can move, zoom in, or rotate the scene for adaptive visualization; 3) An image control panel for data loading and writing controls; 4) A floating 2D overlay visualization panel for visualizing how the tuned model aligns to the image in real time. **b**, BamaPig3D dataset labeling workflow. To obtain fully annotated 3D keypoints for each pig, we first labelled 2D visible keypoints and performed triangulation to obtain visible 3D keypoints (blue). Then, we manually tuned the PIG model to fit image observations and obtain estimated keypoints regressed from the posed mesh (red). Finally, we reserved visible keypoints (blue ones) and fill invisible keypoints with mesh estimated ones (red ones). **c**, Percentage of visible keypoints for different body parts under different camera views. Body part annotation as shown in Supplementary Fig. 4c. Different colors indicate different camera groups. Note that when the cameras go lower, there is a greater probability for head keypoints to be occluded while a lower probability for limbs. This indicates that lower cameras are more suitable for capturing the details of limb motion, while higher ones are more suitable for that of main body motion. **d**, Cross-view visibility study with respect to each keypoint. Data summary shown in **c** and **d** are plotted using the BamaPig3D dataset.

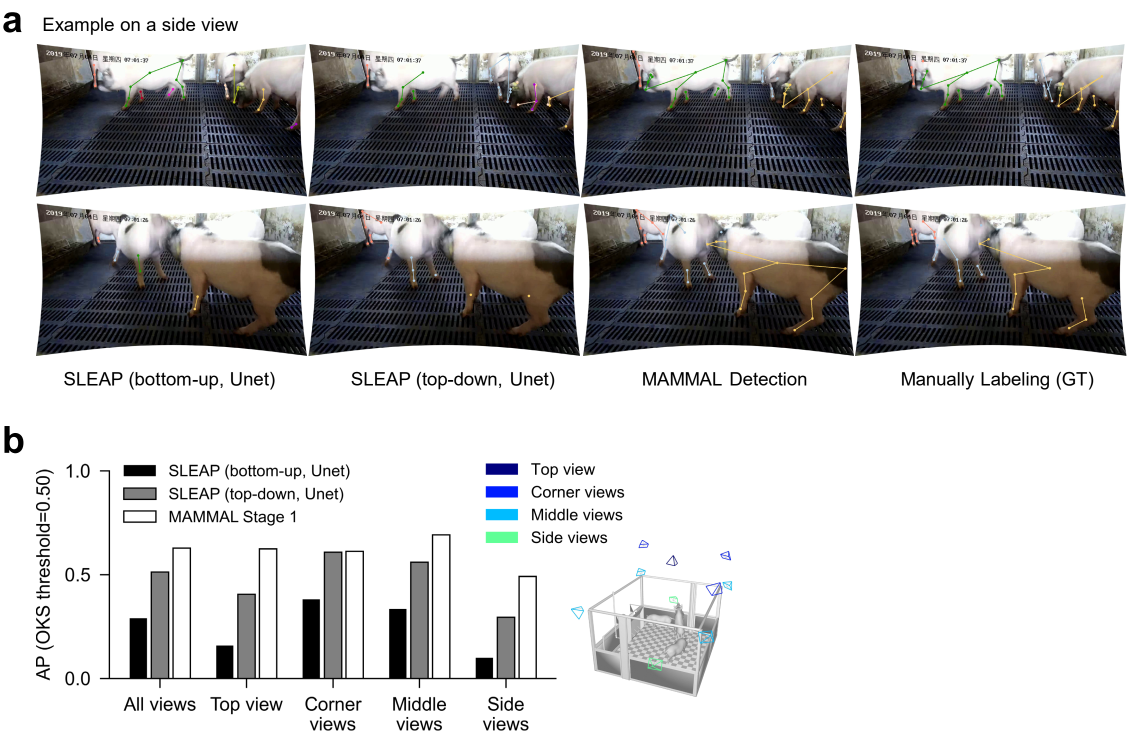

Supplementary Figure 9. Comparisons between MAMMAL Detection and SLEAP. a, Qualitative comparison on a side view on two different time points of BamaPig3D dataset (top row and bottom row). Different colors represent different identities recognized. SLEAP with bottom-up structure and Unet backbone failed to assemble keypoints well due to frequently missing parts (left); SLEAP with top-down structure and Unet backbone failed to identify correct body parts in occlusion cases and failed to recognize keypoints of pigs which occupied large areas of the image (middle left); Our 2D pose detection pipeline succeeded to recognize challenging keypoints (middle right) and was comparable to manually labeling (right) in most cases. **b**, Comparison of 2D pose estimation performance between SLEAP (PMID: 35379947 [https://doi.org/10.1038/s41592-022-01426-1]) and MAMMAL Detection on the 700 manually annotated images of BamaPig3D dataset (70 images per view, 10 views). Both bottom-up and top-down structures of SLEAP with Unet backbone were compared. The comparison metric is average precision (AP) introduced by SLEAP with the threshold for object keypoint similarity (OKS) set to 0.5. The higher AP indicates the better 2D pose estimation performance. Different types of camera views are highlighted using different colors on the right image.

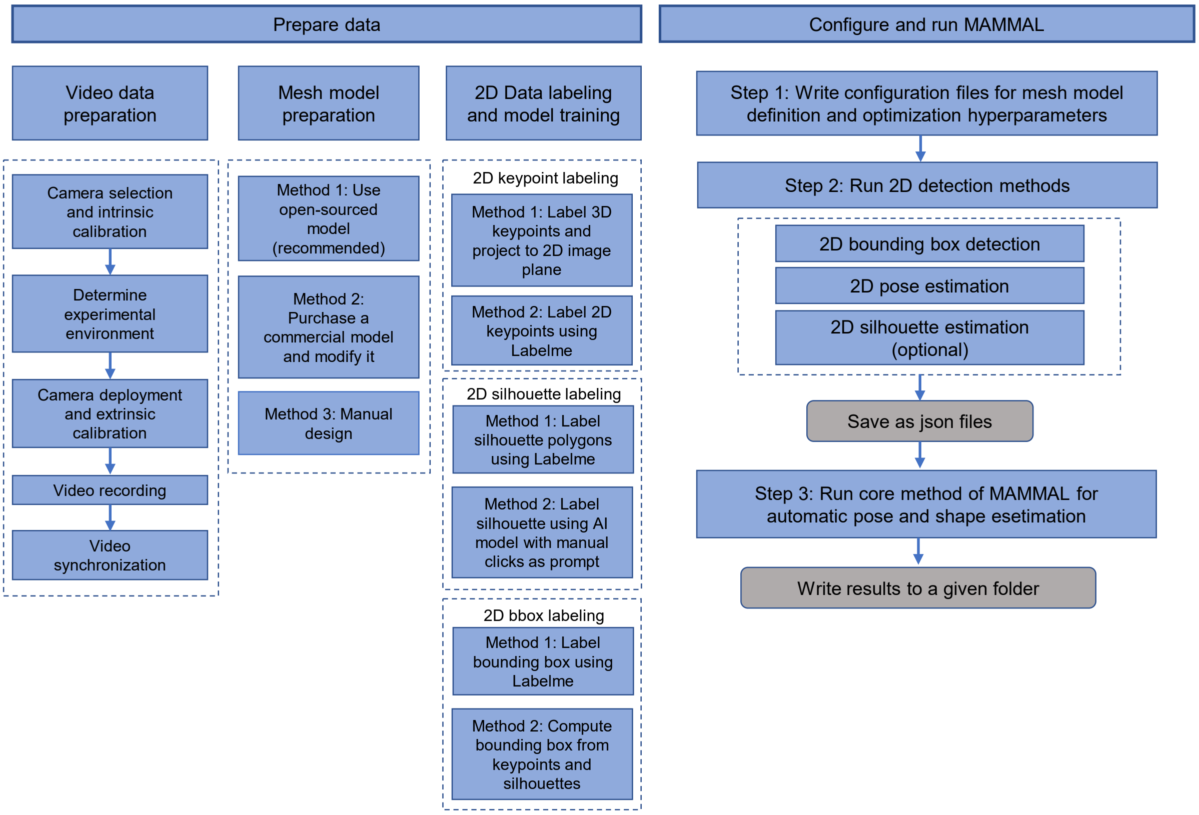

Supplementary Figure 10. The whole pipeline for using MAMMAL on a customized animal motion capture scenario. The whole process of using MAMMAL for animal motion capture can be divided into two parts. The first part is "Prepare data", which means one should prepare video data, mesh model and 2D data labeling for further model training. The second part is "Configure and run MAMMAL", which means one should carefully write the configuration files, run the 2D detection methods and finally run the MAMMAL system.
