# Supplementary material for: Three-dimensional surface motion capture of multiple freely moving pigs using MAMMAL
Source: Nat Commun. 2023 Nov 25;14:7727. doi: 10.1038/s41467-023-43483-w (PMC10673844; doi:10.1038/s41467-023-43483-w)
Supplement: Supplementary file 3 — Description of Additional Supplementary Files [file 41467_2023_43483_MOESM3_ESM.pdf]

Description of Additional Supplementary Files:

Supplementary Movie 1: The PIG model. The first part of this video shows sequences of flexible pig motions produced by 62 joints. The second part of this video compares the motions generated from the full 62 joints and 24 crucial joints. Only subtle differences exist in the ears, toes, and tail, which have limited influence on social behavior analysis.

Supplementary Movie 2: Pipeline of MAMMAL. The full MAMMAL pipeline consists of the following steps: 1) preparing synchronized multiple-view calibrated videos; 2) applying MAMMAL Detection to generate unordered bounding boxes, silhouettes, and keypoints of each individual in each view (stage 1); 3) applying MAMMAL Detection Matching function (stage 2) to match 2D cues to individual animals both spatially and temporally; 4) for each matched pig, applying the MAMMAL Mesh Fitting function (stage 3) to generate a posed PIG model from 2D keypoints and silhouettes; 5) an optional smoothing step is applied to smooth pig poses across the whole sequence.

Supplementary Movie 3: MAMMAL enables animal-scene interaction measurement. MAMMAL enables measurements of animal-scene interactions, including 1) eating behavior related to the feeding area (trough) and 2) drinking behavior related to the drinking area (taps).

Supplementary Movie 4: MAMMAL identifies 8 distinct pig postures. This video shows the 3D pig postures at 8 distinct local density peaks. Since no temporal spectrum was used for clustering, this video only shows static postures instead of dynamic trajectories.

Supplementary Movie 5: MAMMAL enables automatic pig social behavior recognition. This video demonstrates seven types of dyadic social behaviors in a 40-second video. Behaviors are labeled on the left panel. In each label, the left color indicates the active individual, while the right color indicates the passive individual for specific behaviors.

Supplementary Movie 6: MAMMAL tracks multiple pigs with their own identities in a longitudinal study. This video contains four parts. Part 1, the results of the 4 pigs in the BamaPig2D dataset using MAMMAL; Part 2, the results of the same 4 pigs at their older age using MAMMAL; Part 3, the results of 3 pigs; Part 4, the results of another 4 pigs using MAMMAL. MAMMAL can track multiple pigs in a longitudinal study in a non-invasive manner.

Supplementary Movie 7: Comparisons between MAMMAL and Triangulation. The comparison was performed between the 19 keypoints estimated by MAMMAL and those estimated by Triangulation. No temporal smoothing was applied during the comparison. It is shown that MAMMAL always produces full keypoints, while triangulation often loses some keypoints due to poor 2D keypoint detection caused by occlusions or fast motion.

Supplementary Movie 8: Tail motion reconstruction of pigs. Left, the raw video. Middle, 2D pose detection results with tail keypoints. Right, the mesh model overlaid on the raw video.

Supplementary Movie 9: Comparison between MAMMAL and DANNCE-T for single mouse motion capture. We conducted visualized comparison between MAMMAL (bottom) and the temporal version of DANNCE (DANNCE-T, top) on the first 300 frames of the “markerless_mouse_1” sequence proposed by DANNCE. Three out of six views were rendered for comparison. We reduced the original video frame rate from 100 fps to 25 fps to better display the smoothness of the poses. MAMMAL outperformed DANNCE-T in tracking the tail and front paws of the mouse.

Supplementary Movie 10: MAMMAL reconstructs the motion of two Beagle dogs. Top left, the raw video recorded at 120 fps. Top right, detection results of 29 keypoints. Bottom left, the reconstructed dog meshes overlaid on the raw video. Bottom right, 3D rendering of the reconstructed meshes from the top view.
