# Supplementary material for: Three-dimensional surface motion capture of multiple freely moving pigs using MAMMAL
Source: Nat Commun. 2023 Nov 25;14:7727. doi: 10.1038/s41467-023-43483-w (PMC10673844; doi:10.1038/s41467-023-43483-w)
Supplement: Supplementary file 14 — Reporting Summary [file 41467_2023_43483_MOESM14_ESM.pdf]

nature portfolio

Corresponding author(s): Tang Hai, Yichang Jia, Yebin Liu

Last updated by author(s): Oct 22, 2023

Reporting Summary

Nature Portfolio wishes to improve the reproducibility of the work that we publish. This form provides structure for consistency and transparency in reporting. For further information on Nature Portfolio policies, see our Editorial Policies and the Editorial Policy Checklist.

Statistics

For all statistical analyses, confirm that the following items are present in the figure legend, table legend, main text, or Methods section.

n/a	Confirmed	
☐	☒	The exact sample size (*n*) for each experimental group/condition, given as a discrete number and unit of measurement
☐	☒	A statement on whether measurements were taken from distinct samples or whether the same sample was measured repeatedly
☐	☒	The statistical test(s) used AND whether they are one- or two-sided<br>*Only common tests should be described solely by name; describe more complex techniques in the Methods section.*
☒	☐	A description of all covariates tested
☐	☒	A description of any assumptions or corrections, such as tests of normality and adjustment for multiple comparisons
☐	☒	A full description of the statistical parameters including central tendency (e.g. means) or other basic estimates (e.g. regression coefficient) AND variation (e.g. standard deviation) or associated estimates of uncertainty (e.g. confidence intervals)
☐	☒	For null hypothesis testing, the test statistic (e.g. *F*, *t*, *r*) with confidence intervals, effect sizes, degrees of freedom and *P* value noted<br>*Give P values as exact values whenever suitable.*
☒	☐	For Bayesian analysis, information on the choice of priors and Markov chain Monte Carlo settings
☒	☐	For hierarchical and complex designs, identification of the appropriate level for tests and full reporting of outcomes
☒	☐	Estimates of effect sizes (e.g. Cohen's *d*, Pearson's *r*), indicating how they were calculated

Our web collection on statistics for biologists contains articles on many of the points above.

Software and code

Policy information about availability of computer code

Data collection	Custom code was used for all components of the framework of MAMMAL. The key code of MAMMAL written in C++ is released at https://github.com/anl13/MAMMAL_core. Codes for MAMMAL detection are released at https://github.com/anl13/pig_silhouette_det and https://github.com/anl13/pig_pose_det. Code and data related to evaluation and behavior analysis can be linked through https://github.com/anl13/MAMMAL_core. Code for mouse version of MAMMAL can be found at https://github.com/anl13/MAMMAL_mouse. All the code and data can also be accessed at https://doi.org/10.17605/OSF.IO/F6JC5.
Data analysis	Custom code was used for all components of the framework of MAMMAL. The key code of MAMMAL written in C++ is released at https://github.com/anl13/MAMMAL_core. Codes for MAMMAL detection are released at https://github.com/anl13/pig_silhouette_det and https://github.com/anl13/pig_pose_det. Code and data related to evaluation and behavior analysis can be linked through https://github.com/anl13/MAMMAL_core. Code for mouse version of MAMMAL can be found at https://github.com/anl13/MAMMAL_mouse. All the code and data can also be accessed at https://doi.org/10.17605/OSF.IO/F6JC5. Additional commercial or third party software used: numpy 1.17.5, scipy 1.6.0, sklearn 0.24.1, skimage 0.18.1 and matplotlib 3.1.3.

For manuscripts utilizing custom algorithms or software that are central to the research but not yet described in published literature, software must be made available to editors and reviewers. We strongly encourage code deposition in a community repository (e.g. GitHub). See the Nature Portfolio guidelines for submitting code & software for further information.

Data

Policy information about availability of data

All manuscripts must include a data availability statement. This statement should provide the following information, where applicable:

- Accession codes, unique identifiers, or web links for publicly available datasets
- A description of any restrictions on data availability
- For clinical datasets or third party data, please ensure that the statement adheres to our policy

The data for PIG model is provided at https://github.com/anl13/PIG_model. The BamaPig2D and BamaPig3D datasets are at https://github.com/anl13/MAMMAL_datasets. Data for evaluation and behavior analysis are released with the code. All the code and data can also be accessed at https://doi.org/10.17605/OSF.IO/F6JC5. Source data are provided with this paper.

Human research participants

Policy information about studies involving human research participants and Sex and Gender in Research.

Reporting on sex and gender	*Use the terms sex (biological attribute) and gender (shaped by social and cultural circumstances) carefully in order to avoid confusing both terms. Indicate if findings apply to only one sex or gender; describe whether sex and gender were considered in study design whether sex and/or gender was determined based on self-reporting or assigned and methods used. Provide in the source data disaggregated sex and gender data where this information has been collected, and consent has been obtained for sharing of individual-level data; provide overall numbers in this Reporting Summary. Please state if this information has not been collected. Report sex- and gender-based analyses where performed, justify reasons for lack of sex- and gender-based analysis.*
Population characteristics	*Describe the covariate-relevant population characteristics of the human research participants (e.g. age, genotypic information, past and current diagnosis and treatment categories). If you filled out the behavioural & social sciences study design questions and have nothing to add here, write "See above."*
Recruitment	*Describe how participants were recruited. Outline any potential self-selection bias or other biases that may be present and how these are likely to impact results.*
Ethics oversight	*Identify the organization(s) that approved the study protocol.*

Note that full information on the approval of the study protocol must also be provided in the manuscript.

Field-specific reporting

Please select the one below that is the best fit for your research. If you are not sure, read the appropriate sections before making your selection.

[x] Life sciences [ ] Behavioural & social sciences [ ] Ecological, evolutionary & environmental sciences

For a reference copy of the document with all sections, see nature.com/documents/nr-reporting-summary-flat.pdf

Life sciences study design

All studies must disclose on these points even when the disclosure is negative.

Sample size	Totally 17 Bama mini-pigs and 2 Beagle dogs were housed and videotaped for experiments. For demonstrating our software and algorithms, this sample size is sufficient.
Data exclusions	No data was excluded.
Replication	Yes, the algorithms were initially built on four pigs and further validated and repeated on another four pigs unseen by the datasets or the trained model.
Randomization	Data in the proposed datasets was randomly split into training and test sets. Pigs used for experiments were randomly selected.
Blinding	The labeling of the datasets were blind to the algorithms or any other usage of the data.

Reporting for specific materials, systems and methods

We require information from authors about some types of materials, experimental systems and methods used in many studies. Here, indicate whether each material, system or method listed is relevant to your study. If you are not sure if a list item applies to your research, read the appropriate section before selecting a response.

Materials & experimental systems

n/a	Involved in the study
☒	☐ Antibodies
☒	☐ Eukaryotic cell lines
☒	☐ Palaeontology and archaeology
☐	☒ Animals and other organisms
☒	☐ Clinical data
☒	☐ Dual use research of concern

Methods

n/a	Involved in the study
☒	☐ ChIP-seq
☒	☐ Flow cytometry
☒	☐ MRI-based neuroimaging

Animals and other research organisms

Policy information about studies involving animals; ARRIVE guidelines recommended for reporting animal research, and Sex and Gender in Research

Laboratory animals	17 male Bama mini-pigs in total were used for different experiments. Pigs are raised in normal atmospheric temperature. All of the videotaped pigs were at the age between 2 months and 18 months. Additionally, two adult male Beagle dogs were used for demonstration.
Wild animals	No wild animals were used in this study.
Reporting on sex	As a method paper, sex did not influence our algorithm design, therefore we only used male pigs and male dogs for experiments.
Field-collected samples	No field-collected samples were used in this study.
Ethics oversight	Bama mini-pig studies were performed in accordance with an institutional authorization by Institute of Zoology Chinese Academy of Sciences (IOZ-IACUC-2022-185). Beagle dogs studies were approved and performed in the laboratory of Beijing Sinogenetic Biotechnology Co., Ltd.

Note that full information on the approval of the study protocol must also be provided in the manuscript.
